# A 1D model characterizing the role of spatiotemporal contraction distributions on lymph transport

**DOI:** 10.1038/s41598-023-48131-3

**Published:** 2023-12-01

**Authors:** Farbod Sedaghati, J. Brandon Dixon, Rudolph L. Gleason

**Affiliations:** 1 The George W. Woodruff School of Mechanical Engineering, Georgia Institute of Technology, Atlanta, GA USA; 2 The Wallace H. Coulter Georgia Tech/Emory Department of Biomedical Engineering, Georgia Institute of Technology, Atlanta, GA USA; 3 The Wallace H. Coulter Georgia Tech/Emory Department of Biomedical Engineering, Georgia Institute of Technology, 387 Technology Circle, Room 216F, Atlanta, GA 30313 USA

**Keywords:** Computational models, Cardiovascular diseases

## Abstract

Lymphedema is a condition in which lymph transport is compromised. The factors that govern the timing of lymphatic contractions are largely unknown; however, these factors likely play a central role in lymphatic health. Computational models have proven useful in quantifying changes in lymph transport; nevertheless, there is still much unknown regarding the regulation of contractions. The purpose of this paper is to utilize computational modeling to examine the role of pacemaking activity in lymph transport. A 1D fluid–solid modeling framework was utilized to describe the interaction between the contracting vessel and the lymph flow. The distribution of contractions along a three-lymphangion chain in time and space was determined by specifying the pacemaking sites and parameters obtained from experimentation. The model effectively replicates the contractility patterns in experiments. Quantitatively, the flow rates were measured at 5.44 and 2.29 ${\upmu L}/\mathrm{h}$, and the EF values were 78% and less than 33% in the WT and KO models, respectively, which are consistent with the literature. Applying pacemaking parameters in this modeling framework effectively captures lymphatic contractile wave propagations and their relation to lymph transport. It can serve as a motivation for conducting novel studies to evaluate lymphatic pumping function during the development of lymphedema.

## Introduction

The lymphatic system transports interstitial lymph fluid from tissues back to the venous system usually against an adverse pressure gradient. Lymph is collected through primary (initial) lymphatics and is then transported through contractile and collecting (secondary) lymphangions primarily via orchestrated contractions of lymphatic muscle cells. It passes through lymph nodes and ducts before entering the circulatory system. The primary lymphatic vessels absorb interstitial fluid by communicating with the surrounding interstitium through primary lymphatic valves and transport the generated lymph^1^. To achieve this, secondary lymphatics contract autonomously, driving the lymph through one-way valves. This autonomous contractile behavior is governed by various factors, including mechanical stretch of lymphangions, the chemical microenvironment, and electrical conductivity, among others^2–5^. Dysfunction of the lymphatic system leads to lymphedema, a condition characterized by swelling and the accumulation of lymph in a specific limb or region.

Computational models have proven to be useful in characterizing lymphatic contractile behavior and lymph transport^6, 7^. However, much remains unknown regarding the coordination of contractions across lymphangions^8^. Proper timing of contractions between adjacent lymphangions is essential for lymph transport. It is increasingly clear that the timing of lymphatic contractions may be governed by pacemaking cells within the lymphatics. Pacemaking cells serve as the sites of initiation for the spontaneous transient depolarization that drives lymphatic contraction and electrically propagates to adjacent lymphatic muscle cells^9, 10^.

Lumped-parameter models (LPMs) are the most used approach to studying lymphatic mechanics and simulating lymph transport^7, 11^. In such models, the solver takes input of the pressure gradient and contraction frequency of a lymphatic vessel and calculates the transmural pressure and flow. However, these models have some drawbacks. They do not specifically locate the pacemakers, do not consider contraction propagation speed, and do not account for pressure/flow waves. These features, which have been suggested to influence lymphatic pump performance in more complex 3D fluid–structure interaction models, are missing in LPMs^12, 13^. Another limitation of the LPMs is that they do not address the nonlinearity of the balance laws in the algebraic equations. For example, Bertram et al. demonstrated that pressure evenly ramps up along a lymphatic chain, which may not be the case^14^. Furthermore, studying the coordination of contractions in multi-lymphangions is an interesting aspect that is not accessible in the LPMs^15^. Additionally, the lymphatic function is significantly disturbed in damaged lymphatic vessels, where both the contractile and mechanical properties are compromised^16^. To the best of our knowledge, no studies, including the recent 1-D models, have implemented pacemaking activity and contraction propagation speed in their models^17^. To address these issues, a 1-D discretized model would be helpful in quantifying the time and space-dependent physical fields.

As the primary driver of lymphatic muscle cell twitch and contractility, activation function has been widely considered in previous mathematical simulations. However, these simulations typically employ trigonometric functions with a time delay between lymphangions to prescribe contractions along a lymphatic chain^18, 19^. The main challenges in prescribing the activation function lie in the absence of experimental pacemaking metrics, which often lead to conflicting results^20^. For instance, early experimental data on lymphatic contractive coordination along a lymphangion chain indicated that while one lymphangion may start contracting, adjacent ones may be in the diastolic phase, suggesting that contractions are not always coupled^21^. Conversely, it has been demonstrated that adjacent lymphangions are electrically coupled, implying that disturbances in a pacemaker can affect nearby contractile activity^22^.

Furthermore, an ongoing debate surrounds the occurrence of maximum pumping in the absence of time delay between lymphangions^23^, with some proposing that subsequent contractions between lymphangions generate stronger propulsion^19^. McHale et al. suggested that an antegrade pumping is more efficient^24^, while Venugopal et al. argued that the coordination of lymphangions and the direction of contraction propagation minimally affect the pumped flow^23^. Wolf et al*.* highlighted that the speed of contraction waves significantly impacts pumping efficiency^13^. However, their study did not incorporate the coordination of a chain of lymphangions, and pacemakers were not included in the contractions. Based on experimental observations, the delay and opening duration of secondary valves are highly dependent on the activation function^25^, and computational models have suggested that slight changes in the relaxation time during diastole can almost double the mean lymph flow rate^26^. Therefore, there is an urgent need to explore the mechanics of contractions and lymph transport by focusing on pacemaking activity and contraction wave propagation.

Therefore, the objective of this paper is to develop a computational model for studying the mechanics of the lymphatic system and quantifying the impact of abnormal wave propagation on pumping efficiency. This study aims to construct a one-dimensional mathematical model based on the fluid–solid governing equations, incorporating a general activation function. The activation function will be formulated using pacemaking parameters such as initiation sites per length, propagation speed and contraction frequency, and will simulate lymph transport in both wild-type and Connexin-45 knock-out (KO) mice, as reported in existing literature. It has been demonstrated that the deletion of connexin-45 disrupts the coupling of lymphatic muscle cells, ultimately leading to the disruption of contraction wave propagation and abnormal lymphatic function^27–31^. The results obtained from both the WT and KO models will be compared to relevant published data to validate our computational findings.

## Results

### Activation function and lumen contractions

**Figure 1:**
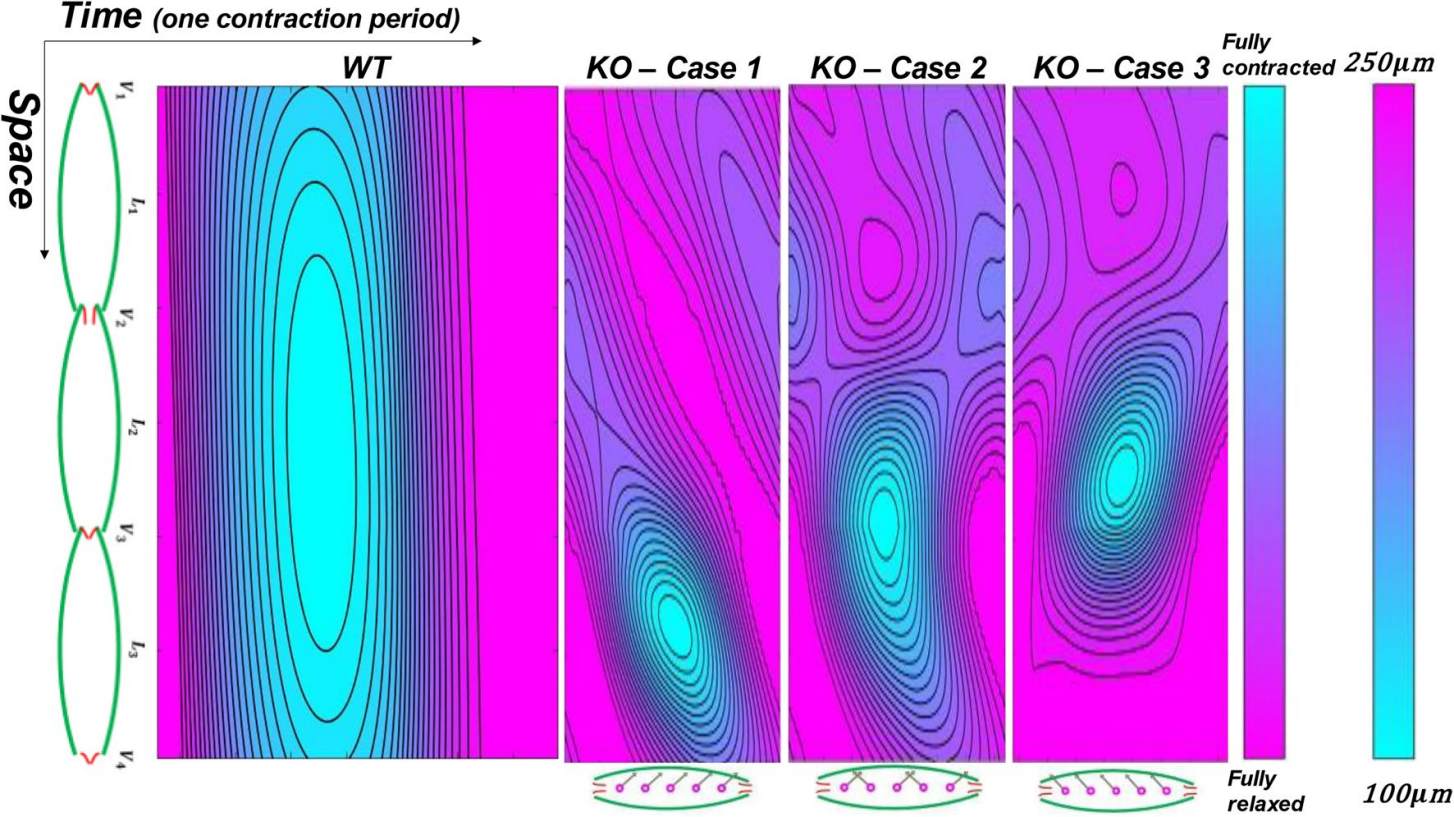
Time–space mapping of contractions of the lymphatic network during a contraction cycle. Contraction cycle of the WT and the KO models are different due to the frequency discrepancy. Contraction patterns of three cases in the KO model are different due to directionality of pacemaking signals. Cyan represents fully contracted state/activated, and purple represents non-contracted state/relaxed.

To visualize the contractions in the time–space domain, we demonstrated the activation function and diameter patterns on a discretized 2-D lymphatic network, along with contraction period mapping. The time–space mapping of contractions illustrates that the peak contraction occurs at different times within the lymphangion chain, depending on both the propagation speed and the proximity of a vessel region to the pacemakers (see Fig. 1). We observed a significant difference in contraction propagation among the four mappings, attributed to the contractility metrics and directionality of the signals. Qualitatively, the model replicates the contractility patterns in experiments. Quantitatively, we introduced a dimensionless parameter called activation integration (AI), which represents the volume integral under the time–space mapping. The values of AI were 189, 51, 69, and 49 for the WT and KO models, respectively. As AI correlates with the applied work by the vessel, one would expect better pump efficiency in the WT model. Despite AI being similar across the three KO models, especially in Case 1 and 3, the pumped lymph flow differed due to the tendency of the valves to stay open at zero pressure gradients. In other words, the forward wave propagation is favored by the valves' tendency to remain open.

**Figure 2:**
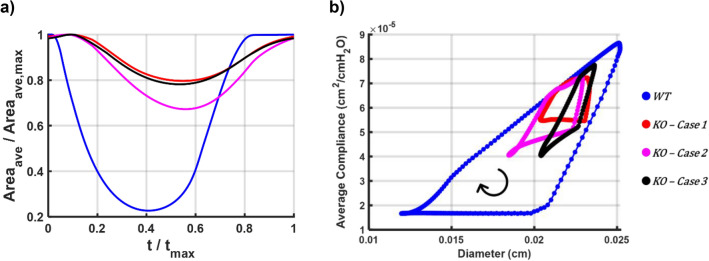
Lumen reduction pattern and compliance-diameter patterns. (**a**) Normalized average area reduction vs. normalized contraction period. Blue corresponds to the WT model showing significant area reduction followed by a relaxation period. Red/purple/black correspond to the KO model showing less remarkable area reduction and missing relaxation period. (**b**) Average compliance vs. diameter pattern during a contraction cycle. Not area reduction and less decrease in the compliance manifests less constriction and stiffening of the lymphatic wall during systole in the KO models.

**Figure 3:**
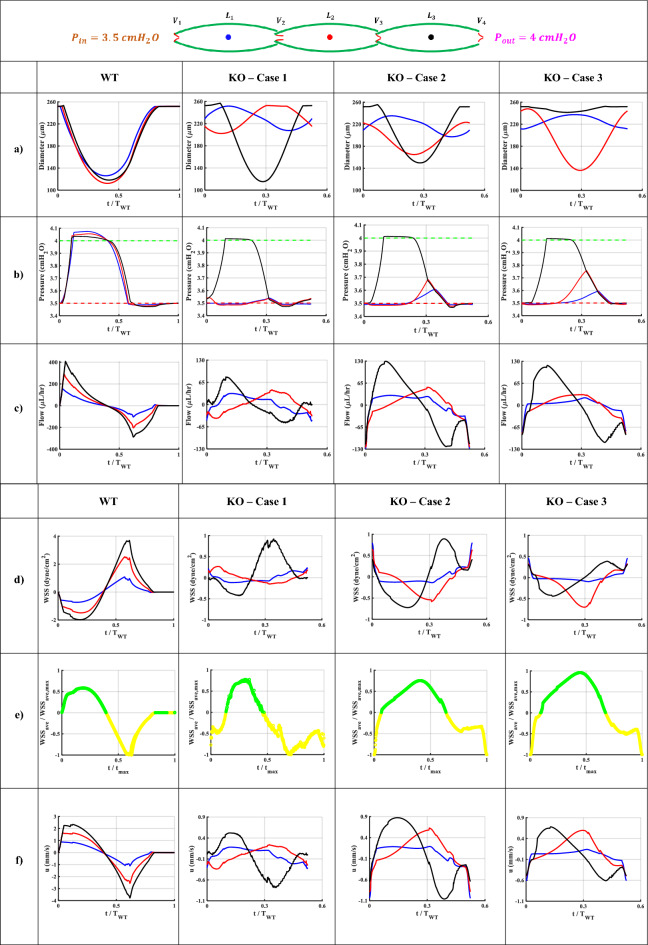

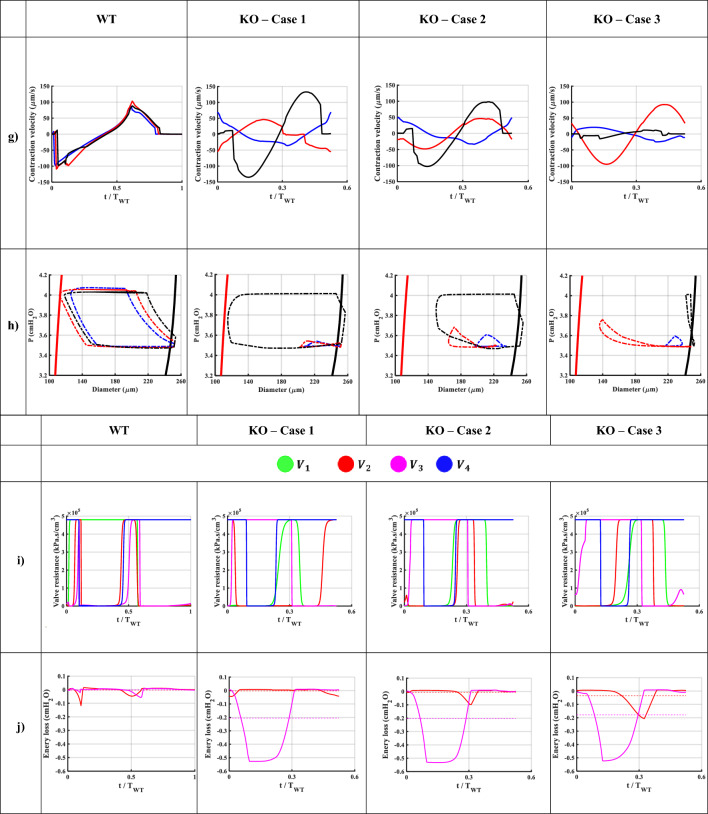
Illustrative simulations of the WT and KO models during a contraction cycle. Results belong to the middle node of the first (blue), second (red), and third (black) lymphangion. (**a**) Diameter patterns represent how contraction waves propagate along the network. (**b**) Pressure pulses and the inlet (red dashed line) and outlet (green dashed line) pressure profiles. (**c**) Lymph axial flow profiles during a contraction cycle. (**d**) Wall shear stress (WSS) patterns exerted on the lymph flow at the wall boundary. (**e**) The normalized average wall shear stress vs. the normalized contraction frequency. Green and yellow show positive and negative values, respectively. (**f**) Lymph flow velocity profiles. (**g**) Radial contraction velocity of the lymphatic vessel. (**h**) Pressure-diameter patterns during a contraction cycle. Dashed black and red lines correspond to the pre- and peak-twitch constitutive data. (**i**) Resistance of the four valves during a contraction cycle. (**j**) The total mechanical energy loss at the middle valves 2 (red) and 3 (purple). The dashed lines represent the mean of each curve. A remarkable energy loss happens in the KO models.

The activation function was utilized to calculate the diameter and compliance of each node. The resulting diameter mapping revealed that diameter patterns were opposite to the activation mappings (Fig. 1); in other words, increased activation led to decreased diameter. The average lumen area contracted by 80% in the WT model, followed by a distinct relaxation period (Fig. 2a). In contrast, in all of the KO models, the area reduction reached a maximum of 30% with no relaxation period. This continuous activation in the KO models may potentially induce fatigue, exacerbating the abnormal contractility. The ejection fraction (EF), which represents the ratio of the volume of fluid dispensed at peak systole to the diastolic volume, was 0.78 for the WT model, while ranging from 0.20 to 0.33 in the KO models. The EF, along with the applied frequency, resulted in a Fractional Pump Function (FPF) of 6 and 4 ${\mathrm{min}}^{-1}$ in the WT and KO models, respectively. Figure 3a illustrates the diameter pattern of the middle node of each lymphangion.

### Pressure patterns

The pressure of the middle node of each lymphangion during a contraction cycle illustrates the propagation of pressure waves along the chains (Fig. 3b). In the WT model, all lymphangions experienced an equivalent pressure rise, matching the total pressure gradient of the chain and the mean pressure in all lymphangions approached the average of ${P}_{in}$ and ${P}_{out}$. However, in the KO models, only the pressure in L3 oscillated between ${P}_{in}$ and ${P}_{out}$, while the pressures in L1 and L2 remained near ${P}_{in}$. This suggests that the primary work performed on the fluid was carried out by a single lymphangion (refer to Table 1).

**Table 1:** Mean pressure in each lymphangion during a contraction cycle. The mean pressure is calculated by averaging the pressure throughout a contraction cycle along the length of a lymphangion.

Model	Mean pressure (${\mathrm{cmH}}_{2}\mathrm{O}$) (average of ${P}_{in}$ and ${P}_{out}$ is 3.75 ${\mathrm{cmH}}_{2}\mathrm{O}$)		
	L1	L2	L3
WT	3.73	3.73	3.74
KO—Case 1	3.50	3.50	3.71
KO—Case 2	3.52	3.52	3.72
KO—Case 3	3.52	3.55	3.73

### Flow patterns

The flow patterns exhibited an impulse in flow at the midpoint of each lymphangion, which coincided with systole in the WT model. This was followed by a dip caused by relaxation and lumen expansion, resulting in flow retracement. Consequently, a net pumped lymph flow of $5.44 {\upmu L}/\mathrm{h}$ was achieved (Fig. 3c). Similar behavior was not observed in the KO models. While a relatively high flow passed through the last lymphangion during systole, the first two lymphangions exhibited opposing (zero or negative) flows. Likewise, during diastole, the flow in L3 was reversed, whereas L1 and L2 had forward flows. The net cycle-averaged lymph flow for Cases 1, 2, and 3 of the KO models was $2.9$, $2.8$ and $1.17 {\upmu L}/\mathrm{h}$, respectively.

The node with the highest flow at each time step in a lymphangion was referred to as the "Bottleneck" point. Upon examining flow profiles along each lymphangion, we observed that the bottleneck node remained stationary in all lymphangions in the WT model during a contraction cycle. This implies that regardless of the phase (systolic or diastolic), a specific region consistently exhibited the highest lymph velocity. In contrast, the bottleneck point was not stable in the KO models, and it moved along the lymphangion. This could provide an additional explanation for the lower net flow observed in the KO cases. At one moment, point A had the maximum flow, while at a later time step, point B located away from A exhibited the maximum flow, resulting in opposing lymph momentum and the disruption of the pressure wave inside each lymphangion.

### Wall shear stress (WSS)

In the WT model, the average, peak systolic and diastolic WSS were 0.08, −2, and 4 $\mathrm{dyn}/{\mathrm{cm}}^{2}$, respectively (Fig. 3d). The manifestation of the sudden pressure wave impulse in the WT model was once again observed in the WSS patterns. Systolic lymph pushing resulted in a sudden drop in WSS followed by a smooth reversal pattern. Additionally, the exerted shear stress on the wall was negative, which is favorable for initiating the next contraction cycle by stimulating the endothelial cells. However, this was not the case in the KO models, where the average WSS was almost zero and the extremum WSS values were lower. In the KO models, WSS varied between −1 to 1 $\mathrm{dyn}/{\mathrm{cm}}^{2}$, and the averages were 0.04, −0.07, and −0.07 $\mathrm{dyn}/{\mathrm{cm}}^{2}$ in Cases 1, 2, and 3 of the KO models, respectively. There was a significant difference in the signal-to-noise ratio (SNR) of the WSS between the WT model (1.24) and the KO models (0.88, 0.69, and 0.70), indicating the noisiness in the WSS in the KO models. In other words, due to the internal interaction between the pressure waves, it is possible that the lymphatic endothelial cells experienced high shear disturbances. Given that WSS has been hypothesized to be an important mechanical regulator in assisting the control of lymphatic contraction, the noisy WSS fluctuations in the KO models could potentially compromise pumping efficacy^32–35^.

Despite the average WSS values across the cycle, the patterns depicted in Fig. 3e reveal the symmetrical nature of $WS{S}_{ave}$ during a contraction. The WT model exhibited nearly identical shear stress on the vessel wall during systole and diastole. However, in the KO models, this symmetry was absent, leading to disorganized shear forces, particularly during diastole. Moreover, abrupt spikes in early systole and late diastole were observed.

### Lymph velocity

In the WT model, a clear systolic peak velocity with the maximum velocity in the last lymphangion was followed by a reduction in velocity, with the maximum reverse velocity occurring at the same location (Fig. 3f). This indicates that the activation function amplified the momentum along the vessel during each propulsion. In the KO models, it was generally observed that the lymph velocity in the first two lymphangions partially opposed that of the last lymphangion during both systole and diastole. It is noteworthy that in all KO models, the peak systolic and diastolic velocities did not exceed 0.9 and −1 mm/s, respectively, whereas in the WT model, they reached up to 2.4 and −3.8 mm/s. The velocity profiles were also out-of-phase in the KO models, and the systolic and diastolic phases of contraction were indistinguishable in the first two lymphangions. Additionally, in the WT model, there was a resting state when the net lymph velocity was zero in all lymphangions. However, in all the KO models, the contraction started while a negative lymph velocity counteracted some of the contraction energy to overcome the backward momentum, resulting in a loss of pumping efficiency.

### Contraction velocity

In the WT model, the lymphangions contracted almost simultaneously at a velocity of $100 \mu m/s$ followed by a similar diastolic velocity (Fig. 3g). The contraction occurred within a short period of time (< 50 ms), indicating that the vessel generated significant momentum to propel the lymph. Additionally, the impulse of the vessel wall was transmitted to the valve leaflets, and as there was no opposing pressure pulse, this triggered the valves to open during the systolic phase. In Case 3 of the KO model, the middle lymphangion exhibited the highest contraction velocity, while in Case 1 and 2, the highest radial velocity occurred in the last lymphangion. Interestingly, the peak systolic and diastolic wall velocities were approximately $100 \mu m/s$ in all models. In other words, despite the relatively high velocity at which the vessel wall contracted in the KO models, it did not generate enough propulsion force to move the lymph due to the asynchrony of the contractions.

### Pressure–diameter (P–D) curves

Pressure-diameter loops during the pumping cycle of the WT model demonstrated a distinct pattern on the P–D plane, delineating the trajectory between the pre- and peak-twitch curves. However, the corresponding curves of the KO model exhibited two significant dysfunctions (Fig. 3h). Firstly, the patterns were not clearly discernible, and the diameters experienced significant disturbances instead of contracting smoothly, lacking a coherent circular pattern on the P–D plane. Secondly, in the KO models, the pressure within each lymphangion remained close to the inlet or outlet pressure values, failing to experience the complete pressure gradient.

### Valve status

The valve mechanics were assumed to be identical in each case, and therefore all valves operated in each of the simulations. However, the order and duration of openings/closures varied (Fig. 3i). In the WT model, the contraction signal began with sequential closure of Valves 1 and 2, followed by the opening of Valves 3 and 4, and then Valve 2. Once the contraction subsided and the pressure dropped, the downstream valves closed. During late diastole, only Valve 4 was closed while all other valves remained open, preparing for the next cycle. In the KO models, during systole, we expected only the first valves to be closed to aid lymph propulsion. However, Valve 3 was also closed, indicating that while some lymph was discharged from the last lymphangion, lymph was trapped in the first two lymphangions and not transported to the third lymphangion. During diastole, the downstream valves were closed, and Valve 1 was open as expected to provide lymph for the next cycle. Valves 2 and 3 were partially closed, implying that less lymph volume could enter the vessel during diastole. Thus, the valve state alone suggests that the net pumped lymph was significantly compromised in the KO models, both because less lymph was propelled during systole and less volume was stored during diastole. Therefore, although all valves operated in the KO models, local pressure opposition caused backflow in the valves, resulting in a significant reduction in net forward flow.

### Compliance–diameter (C–D) curves

Compliance refers to the ease of inflating a vessel to a specific radius and is inversely correlated with stiffness. During systole, when the vessel wall contracts due to lymphatic muscle cells, we anticipate a decrease in vessel compliance. In essence, the lymphatic muscle cells transform into stiffer fibers to fortify the vessel, strengthening its walls and preventing an increase in tube caliber. Instead, they aid in the forward movement of lymph. Three compliance phases were identified during a pumping cycle (see Fig. 2b). In early systole, both the diameter and compliance decrease, followed by an iso-compliant phase where the diameter continues to decrease while compliance remains constant. Subsequently, both the diameter and compliance increase, returning to their pre-twitch states. The iso-compliant phase illustrates that, under normal conditions, muscle cells twitch synchronously and remain activated as the lumen closes. However, in the KO models, the three phases were hardly distinguishable. Despite the diameter decreasing, the compliance did not fluctuate nearly as much as in the WT case. In other words, regardless of the overall health of the muscle cells, due to abnormal contractility, the vessel walls did not stiffen sufficiently to propel the lymph.

### Energy loss at valves

The average energy loss in the WT model was less than $0.1 cm{H}_{2}O$. This value remained consistent for Valve 2 in the KO models. However, Valve 3 exhibited a remarkable energy loss of $0.2 \mathrm{cm}{\mathrm{ H}}_{2}\mathrm{O}$ (Fig. 3j). It's important to note that a $0.2 \mathrm{cm}{\mathrm{ H}}_{2}\mathrm{O}$ energy loss is equivalent to 40% of the total pressure head that the vessel pumped against. In other words, due to abnormal contractility, the vessel must work harder to successfully discharge the interstitial fluid, overcoming both the total pressure gradient and the energy losses at the valves. The pressure loss at the valves, along with potential flow disturbances on the leaflets, may drive additional changes in the leaflet morphology through remodeling mechanisms.

## Discussion

Regarding modeling outputs, Fig. 2a illustrates the reduction in luminal area, while Fig. 3a depicts diameter fluctuations. These figures demonstrate how changes in pacemaking and contraction wave propagation can impact radial contraction patterns and lymph transport. The reduction of vessel caliber during systole in the WT model (~ 50%) aligns with ex-vivo data^9^. Moreover, the EF and FPF values for both the WT and KO models closely resemble previously published experimental results^2, 29, 36^. The contraction velocity (~ 100 ${\upmu m}/\mathrm{s}$) calculated in the simulations closely matches the values reported by Zhang et al.^37^.

In the WT model, the average and peak flow rates were 5.44 and 400 ${\upmu L}/\mathrm{h}$, respectively, while they ranged from 1.17 to 2.9 and from 80 to 150 ${\upmu L}/\mathrm{h}$ in the KO models. Dixon et al*.* and Zawieja reported the average and peak flow rates of 14 ${\upmu L}/\mathrm{h}$ and 100 ${\upmu L}/\mathrm{h}$, respectively, occurring in either the WT or KO models^36, 38^. Dixon et al*.* experimentally estimated the peak reversal lymph velocity ranging from −1 to 7 $\mathrm{mm}/\mathrm{s}$, while the model estimated a range of −4 to −1 $\mathrm{mm}/\mathrm{s}$^38^. Additionally, Zawieja estimated the peak lymph velocity to be between 2 and 9 $\mathrm{mm}/\mathrm{s}$^36^. In an in-vivo study, the peak forward lymph velocity was measured to be 2 to 3 $\mathrm{mm}/\mathrm{s}$; and in the WT model, the calculated peak forward lymph velocity was approximately 2 $\mathrm{mm}/\mathrm{s}$^16^. Furthermore, Rahbar et al*.* reported lymphocyte velocity and average flow rates ranging from 0.1 to 1.8 $\mathrm{mm}/\mathrm{s}$ and 6 ${\upmu L}/\mathrm{h}$, respectively. Although the nature of abnormality differs in their study, the range of lymph velocity and flow covers the estimated values from both the WT and KO models^39^. Interestingly, the peak diastolic negative velocity is higher than the systolic positive velocity in all models, while the net pumped flow remains positive. This highlights the importance of measuring the area and velocity profile to accurately calculate the flow rate, a feat that has only been achieved using Doppler OCT to date^40, 41^.

We conducted a sensitivity study on the experimental data used in the simulations to evaluate the impact of variations in frequency and number of initiation sites on the model outcomes. We employed the mean values of each parameter as specified in the experimental paper. To explore the effects of experimental method uncertainty on lymph flow, we conducted simulations using two different combinations of frequency and number of pacemakers obtained from the experiments. The results showed that the mean lymph flow could vary by up to 45% when the contraction frequency was set to the minimum measured value, such as 14.5 for the KO scenario. However, this variability was not observed for the number of pacemaking sites. Instead, the mean lymph flow increased from the minimum number of pacemaking sites to the mean values, and then decreased, displaying an inconsistent pattern.

WSS is believed to influence lymphatic contractility due to the sensitivity of lymphatic endothelial cells to changes in WSS and their interaction with lymphatic muscle cells. Like arteries and veins, the magnitude and smoothness of WSS oscillations can potentially affect lymphatic endothelial cell physiology. However, the impact of WSS-induced changes on lymphatic contractile function is not well understood. In our model, the WSS values are partially comparable to those reported by Mukherjee et al*.*, especially when considering differences in vessel diameters and the imposition of high WSS^35^. Their study showed a mean WSS of approximately 0.6 dyn/cm^2^ with a peak of 8 dyn/cm^2^, while our model calculated values of 0.17 and 10–20 dyn/cm^2^. Another study by Zawieja et al*.* demonstrated mean WSS values of 0.4 to 0.6 dyn/cm^2^ with a peak of 3 to 10 dyn/cm^2^, which closely aligns with our model calculations^36^. Furthermore, Wilson et al*.* and Dixon et al*.* estimated an average WSS of 0.64 dyn/cm^2^, Kornuta et al*.* reported values ranging from 0 to 2 dyn/cm^2^, and Rahbar et al*.* stated 0.15 dyn/cm^2^ in control cases^38, 39, 42, 43^. Thus, collectively, the model’s WSS calculations fall within the ranges of those reported in the literature. It is worth noting that since lymphatic vessels experience both forward and reverse flows, the average WSS could be two orders of magnitude smaller than the peak systolic and diastolic values. This indicates that any inaccuracies or noise in measuring average WSS using experimental methods can lead to erroneous readings.

P–D patterns exhibit similarities to experiments in terms of the sequence of diameter reduction and pressure rise^44^. Chaotic P–D patterns in the KO models can serve as a visual tool to demonstrate abnormal contractility in lymphatic vessels. Reduced pressure rises in the KO models indicate that certain lymphangions may experience minimal pressure pulses during contractions, leading to further impairment in contractility due to pressure-contraction couplings. Moreover, P–D patterns in the WT model indicate that utilizing the weighted average of diameters, derived from constitutive models, along with an activation parameter, is a suitable alternative to solving the constitutive equation within the 1D model. Additionally, by employing a correction factor proposed by Razavi et al., one can easily scale the pre- and peak-twitch curves to prescribe the mechanical response under a disturbed chemical environment^45^.

The results become even more intriguing when compared to the data on injured lymphatic vessels that underwent remodeling and exhibited disrupted mechanical properties and contraction parameters^6^. If the knockout (KO) model is analogous to the wounded case presented in their study, the contractile frequency of the remodeled vessel for a pressure gradient of 0.5 ${\mathrm{cmH}}_{2}\mathrm{O}$ is 2–3 times higher than that of the control case, which aligns with our findings. The KO model successfully replicated the computationally calculated decrease in lymph flow (~ 2.5 ${\upmu L}/\mathrm{h}$) observed in their in vivo data.

The current model could serve as a valuable tool for studying lymph transport in conjunction with an imaging technique. In other words, contractile metrics obtained from online time–space diameter mappings should be utilized to estimate pressure, flow, and other relevant parameters of a lymphatic specimen. Although the experimental data were employed to construct the model, there is still an urgent need to extensively quantify the lymph velocity profile non-invasively, by using NIR imaging, DOCT techniques, or other methods^40, 46^. It is important to note that this model focused on contractions, assuming that the wall mechanics and valve behavior remained unchanged in abnormal cases. Also, due to the lack of experimental measurements, these couplings were not utilized in this study^47, 48^.

Lymphedema is often accompanied by the growth and remodeling of lymphatic vessels, which involve physical forces exerted on the vessels, affecting vessel behavior on short- and long-time scales^11^. Experimental studies have demonstrated such coupling mechanisms by monitoring contraction outcomes in a controlled mechanical environment^8, 35, 39, 42, 45, 49–52^. However, existing LPMs fail to accurately address the pressure/WSS and contraction couplings due to variation of shear stress along the length of the lymphangion caused by local pacemaking activities^52^. In our model, we decouple contractions from the pressure and WSS effects, and the main driving factor of contractions is an activation function chosen to match representative experimental contractility data from the literature. To enhance the model, it is ideal to incorporate pressure/WSS and contraction couplings to provide feedback for regulating contractions^6, 11^.

It is evident from the model that while the pressure does not undergo significant changes within each lymphangion, the WSS exhibits a varying pattern even along a single lymphangion. This observation indicates that the WSS changes non-linearly along the lymphangion suggesting that the linear distribution of physical parameters within the lymphangions may not be a realistic assumption.

Studies have shown that imposed flow inhibits active pumping in lymphatics^53^. Further investigation could examine the impact of axial pressure gradient and number of lymphangions on pump efficiency^54, 55^. Additionally, the current model could address the effect of contractility on passive and active lymph transport^56^. Other relevant studies related to lymphedema could explore topics such as abnormal wall mechanics^6^, leakiness of secondary valves^57^, and leakiness of primary valves^58^. Conducting a parametric study similar to Jamalian et al*.*^7^, would be an interesting direction to investigate. Another potential avenue is to include the initial lymphatics and explore the "Guyton Suction Theory" regarding the absorption of lymph from the interstitium, which poses a challenging question^59^. The compromised lymph absorption in lymphedema, an intriguing subject addressed in a few studies, could also be incorporated into the model^58, 60, 61^.

A one-dimensional mathematical model, combined with contractility metrics derived from experimental data of wild-type (WT) and knockout (KO) mouse lines, was utilized to examine the mechanical behavior and pumping function of a lymphatic vessel under both healthy and Cx45 KO condition^29^. Additionally, the model provides estimates for mechanically related parameters that are challenging to measure experimentally. These estimates can facilitate parametric studies and investigations into the impact of mechanical properties on lymphatic function and the progression of lymphedema^62^. Furthermore, the model demonstrates that a comprehensive understanding of pump efficiency in lymphatic vessels cannot be achieved by analyzing isolated aspects of mechanical behavior alone. For instance, the model reveals that a high contraction velocity and functioning valves do not necessarily guarantee efficient lymph transport. Disparities among KO models suggest that the directionality of pacemaking signals plays a role, with antegrade contraction propagation appearing to be more efficient in lymph transport, aligning with the accepted paradigm^24^.

Lastly, it is important to mention that this study did not specifically examine the probability of contraction patterns in a lymphatic vessel. Investigating the likelihood of dominant pacemaking locations would require a separate set of simulations^10^. As of now, there is no experimental data available on the contribution of each pacemaking location to the resulting contraction magnitude. Consequently, we have not included illustrative results pertaining to this aspect. Nevertheless, exploring these factors could provide valuable insights and enhance our understanding of lymphatic contractions in future research.

## Methods

**Figure 4:**
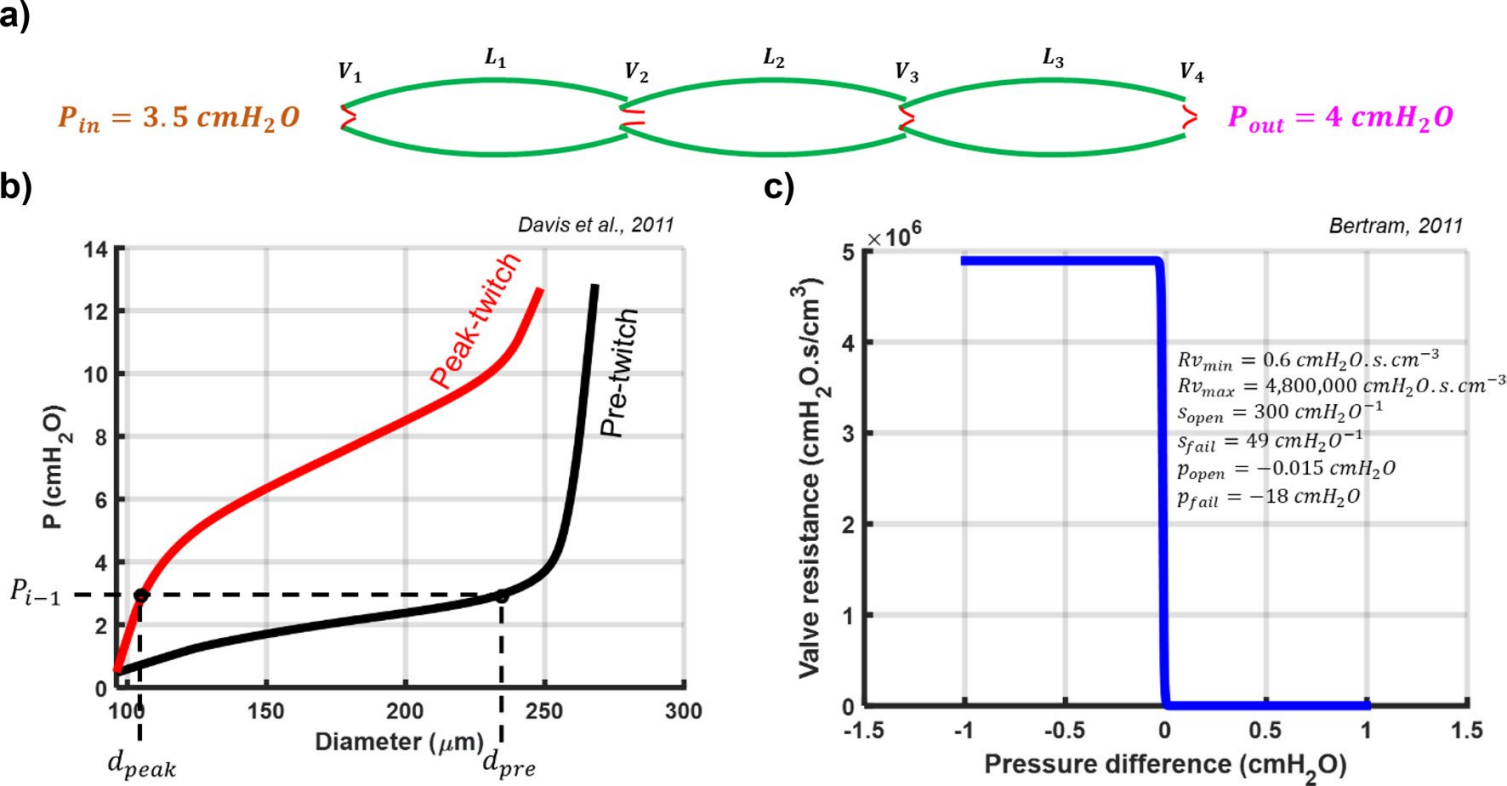
Model representation and constitutive data. (**a**) Schematic representation of the lymphatic network including three lymphangions and four secondary valves. (**b**) Pressure-diameter data of the mesentric lymphatic vessel from male Sprague–Dawley rats^1^. Black and Red represent pre- and peak-twitch data in a inflation test. *dpre* and *dpeak* correspond to pressure from the previous time step. (**c**) Sigmoidal curve of secondary valve resistance vs. pressure difference^2^.

Based on existing experimental data in the literature^29^, we constructed a model of a lymphatic vessel comprising three lymphangions and four secondary valves (Fig. 4a). In all illustrative simulations, the inlet and outlet pressures were set at $3.5$ and $4 {\mathrm{cmH}}_{2}\mathrm{O}$, respectively.

### Theoretical framework

#### 1D fluid mechanics framework

Each lymphangion is modeled as a tapered, elastic tube within a one-dimensional pulsatile fluid flow framework. The principles of mass conservation and linear momentum dictate that,

1 $$C\left(z,t\right)\frac{\partial P\left(z,t\right)}{\partial t}+\frac{\partial Q\left(z,t\right)}{\partial z}=0\,\mathrm{ and }\frac{\rho }{A}\frac{\partial Q}{\partial t}+\frac{\partial P}{dz}=\frac{\tau }{A}-(1+{\delta }_{s})\frac{\rho }{A}\frac{\partial }{\partial z}\left(\frac{{Q(z,t)}^{2}}{A(z,t)}\right)$$

In the given context, $A(z,t)$ represents the instantaneous cross-sectional area of the lumen, $P\left(z,t\right)$ denotes the luminal pressure, $Q\left(z,t\right)$ represents the volumetric flow rate, and $C\left(z,t\right)$ represents the area compliance, given by the expression $\partial A(z,t)/\partial P\left(z,t\right)$. Here, $z$ denotes the direction along the vessel axis, while $t$ refers to time. Other variables in the equation include $\rho$ which is the blood density, and $\tau$ represents the frictional force per unit length. The velocity profile, ${u}_{z}\left(d,z,t\right)$, is prescribed at any given instant $t$ and location $z$ as

2 $${u}_{z}\left(d,z,t\right)=\overline{u }\left(z,t\right)\frac{\zeta +2}{\zeta }\left[1-{\left(\frac{d}{{d}_{i}}\right)}^{\zeta }\right]$$

where $\overline{u }\left(z,t\right)$ is the mean velocity defined as the ratio of $Q(z,t)$ to $A\left(z,t\right)$, $\zeta$ is a constant that determines the shape of the velocity profile, $d$ is the radial coordinate, and ${d}_{i}$ represents the lumen diameter. By utilizing the Naiver-Stokes equations and integrating Eq. (2), the viscous force can be calculated as follows:

3 $$\tau =-2\left(\zeta +2\right)\mu \pi \frac{Q\left(z,t\right)}{A\left(z,t\right)}$$

where $\mu$ is the lymph viscosity. This framework is explained in detail in a previous study^63^.

#### Solid mechanics

To proceed, Eq. (1) require information on the cross-sectional area, $A\left(z,t\right)$, and compliance $C\left(z,t\right)$ at each time ($t$) and location ($z$), given the applied loads ($P\left(z,t\right)$) and the activation state along the lymphangion wall. To characterize the behavior of the wall, we utilize data from Davis et al.^25^. Figure 4b depicts the mechanical states before and at the peak twitch. The pre-twitch represents the dilated configuration when muscle cells are fully relaxed, while the peak-twitch state corresponds to the muscle cells’ peak contractile state.

To determine the diameter for a given pressure and muscle activation, we utilize a weighted-average of pre- and peak-twitch diameters where the weight function is a general activation function, namely,

4 $$d\left(z,t\right)=\left({d}_{peak}-{d}_{pre}\right){t}_{act}+{d}_{pre}$$

where ${d}_{pre}$ and ${d}_{peak}$ represent the pre-twitch and peak- twitch diameters at the pressure from the previous time step of the node, and ${t}_{act}$ is the contraction parameter ranging from 0 and 1. Although the equation is not explicitly a function of space ($z$) and ($t$), as the pre- and peak-twitch diameters change at each node based on previous pressure and ${t}_{act}$ changes both temporally and spatially, the overall function is implicitly a function of $z$ and $t$.

In contrast to employing a known constitutive law for the active and passive mechanical response and solving a highly non-linear inverse problem regarding geometry (i.e., diameter) while considering applied loads (i.e., pressure) and active and passive material properties, Eq. (4) enables an approximate determination of the diameter at each time point and location, with reduced computational cost.

Area compliance may be expressed as,

5 $$C=\frac{\partial A}{\partial P}=\frac{\partial A}{\partial d}\frac{\partial d}{\partial P}=\frac{\partial d}{\partial P}\frac{\partial }{\partial d}\left(\frac{\pi }{4}{d}^{2}\right)=\frac{\pi }{2}d\frac{\partial d}{\partial P}$$

Thus, at pre- and peak-twitch contraction states we have

6 $${C}_{pre}=\frac{\pi }{2}{d}_{pre}{\left(\frac{\partial d}{\partial P}\right)}_{pre}$$

7 $${C}_{peak}=\frac{\pi }{2}{d}_{peak}{\left(\frac{\partial d}{\partial P}\right)}_{peak}$$

where ${C}_{pre}$ and ${C}_{peak}$ represent the compliances of pre-stretch and peak-stretch at ${d}_{pre}$ and ${d}_{peak}$, respectively. Also, ${\left(dD/dP\right)}_{pre}$ and ${\left(dD/dP\right)}_{peak}$ denote the slopes of the pressure-diameter curves. Similar to diameter, in order to estimate the compliance, we let

8 $$C\left(z,t\right)=\left({C}_{peak}-{C}_{pre}\right){t}_{act}+{C}_{pre}$$

#### Valve mechanics

Lymphangions are defined as a segment of a lymphatic vessel bounded between two adjacent pairs of valve leaflets, which function as one-way valves and aid in the forward propulsion of lymph within the network. Due to the small size of these vessels, extracting a valve and measuring the mechanical properties of the leaflets is challenging. Consequently, several experimental studies have been conducted to quantify the mechanics of secondary valves under various pressure gradients. These studies generally propose a sigmoidal pressure-flow relationship. In our study, valve gating is solely influenced by pressure waves within the lymphangions, and the valves' actions are not directly governed by external factors. The only assumption made regarding valve behavior is the valve equation. We adopt the valve behavior introduced by Bertram et al.^19^,

9 $${R}_{v}={R}_{{v}_{min}}+{R}_{{v}_{max}}\left(\frac{1}{1+{e}^{-{s}_{o}\left(\Delta p-{p}_{o}\right)}}+\frac{1}{1+{e}^{-{s}_{f}\left(\Delta p-{p}_{f}\right)}}-1\right)$$

The parameters introduced in Eq. (9) are provided in Fig. 4c.

#### Spatiotemporal activation function

Pacemaking activity is a crucial feature of lymphatic contractility. Numerous studies have demonstrated that specific locations within lymphatic vessels generate contractions, and these locations can vary from one lymphangion to another. These specific sites are referred to as “pacemaking sites” or “pacemakers”. In addition to identifying these cells, various metrics are typically measured to study the contractions, such as contraction frequency and propagation speed and pacemaking distance^9, 10^. These studies reveal that contractions exhibit wave-like behavior, highlighting the necessity of employing a 1-D modeling approach to investigate the lymphatic system. This approach enables the quantification of contraction propagation along a vessel. Building on an experimental study by Zawieja et al., it has been observed that contractions within a lymphatic network propagate both temporally and spatially at a certain speed. These waves have been shown to transmit between adjacent lymphangions via gap junctions^21^. So, the spatial–temporal propagation of contractions forms the fundamental concept of our model.

The temporal and spatial propagation of contractions is quantified using a 2-D bivariate Gaussian function, which has the general form of

10 $$f\left(z,t\right)=\frac{1}{2\pi {\sigma }_{z}{\sigma }_{t}\sqrt{1-{\rho }^{2}}}{e}^{\left[-\frac{1}{2\left(1-{\rho }^{2}\right)}\left(\frac{{\left(z-{\mu }_{z}\right)}^{2}}{{\sigma }_{z}^{2}} - \frac{2\rho \left(z-{\mu }_{z}\right)\left(t-{\mu }_{t}\right)}{{\sigma }_{z}{\sigma }_{t}} + \frac{{\left(t-{\mu }_{t}\right)}^{2}}{{\sigma }_{t}^{2}}\right)\right]}$$

where ${\mu }_{z}$ represents the spatial location of a pacemaker determined by pacemaking distance, ${\mu }_{t}$ denotes the temporal location of a pacemaking signal, and ${\sigma }_{z}$, ${\sigma }_{t}$, and $\rho$ are the spatial, temporal, and correlated standard deviations, respectively. In this function, the center of a distribution is positioned at the pacemaker site (${\mu }_{z}$) when it is expected to generate a signal (${\mu }_{t}$). The temporal standard deviation (${\sigma }_{t})$ is associated with the rate at which the contraction wave diminishes over time. Physically, this parameter may be influenced by the speed at which ion channels are excited, how rapidly gap junctions transport ions, and how quickly this translates into force generation. The spatial standard deviation (${\sigma }_{z})$ is linked to the distance traveled by the contraction wave along the length of a chain of lymphangions. Essentially, this parameter simulates cellular resistance and the ease with which cells transmit the contraction wave.

**Figure 5:**
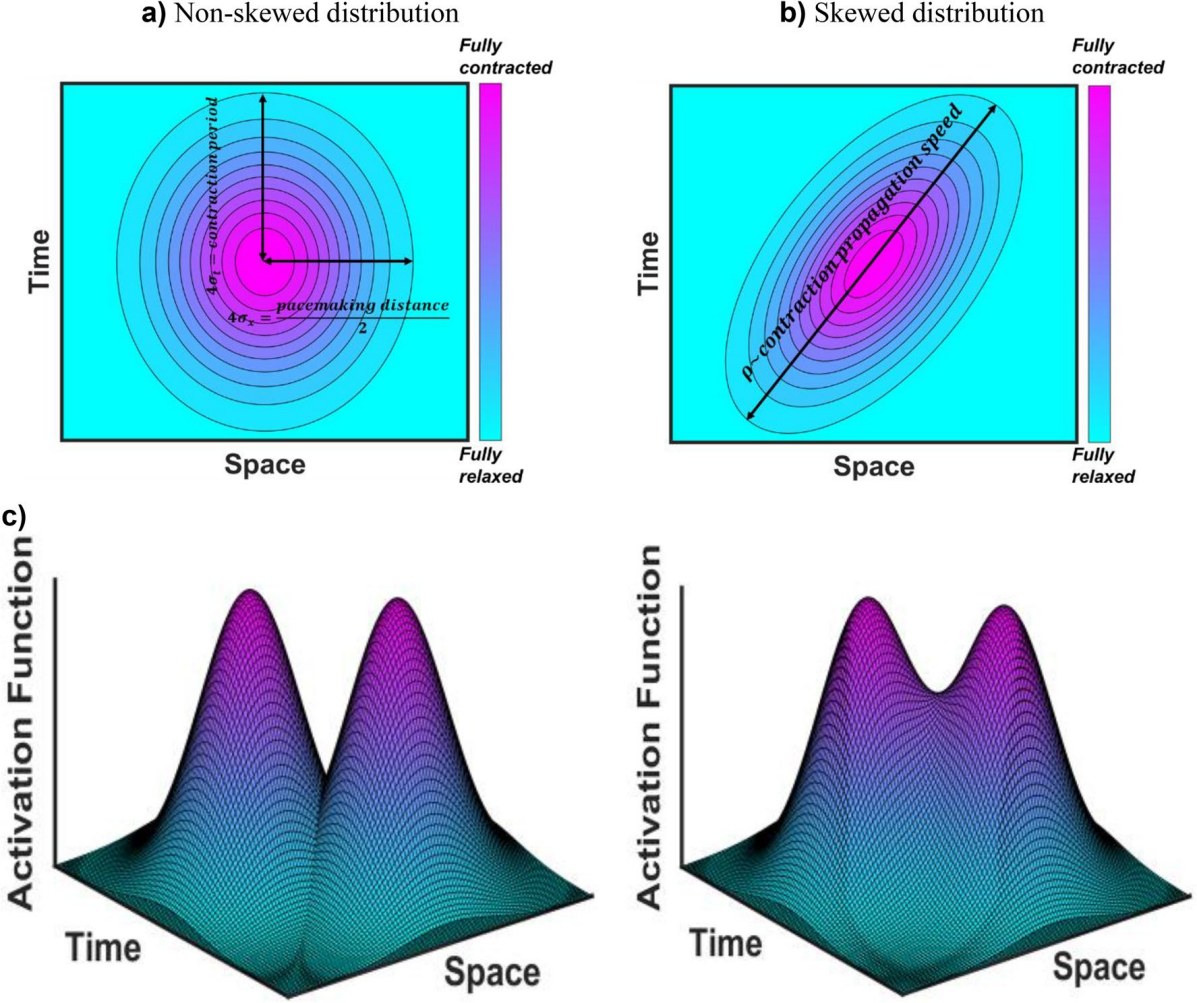
Schematic representation of standard deviations of Gaussian function and “summation” concept. (**a**) Spatial and temporal standard deviations are correlated to the pacemaking distance and contraction frequency, respectively. Zero correlation results in a symmetric Gaussian distribution or non-skewedness which happens in high contraction propagation speed. (**b**) Low contraction propagation speed results in a significant correlation and skewedness of the Gaussian distribution. (**c**) Using the concept of “summation” to generate the overall activation function based upon individual Gaussian distributions at pacemakers.

To determine the pacemaking parameters, we assume that contractions must decay between any two pacemakers in a contraction period timeframe. Spatially, there should be two decays between any two pacemakers. Mathematically, one decay corresponds to approximately 4 standard deviations, denoted as ${\sigma }_{z}={L}_{pacemaker}/8$. Similarly, there should be one decay within a contraction period, resulting in a standard deviation of ${\sigma }_{t}={T}_{contraction}/4$ (refer to Fig. 5a). $\rho$, known as the “correlation”, represents the contraction speed and describes how the activation distribution is skewed in the time and the space domains. The minimum value of $\rho$ is 0, indicating a high contraction propagation speed and a non-skewed distribution. The maximum limit of $\rho$, derived from the definition of the Jacobian of the covariance matrix, is $\sqrt{{\sigma }_{z}{\sigma }_{t}}$. This occurs when the propagation speed is low, and the distribution is highly skewed in the space–time domains (see Fig. 5b).

To construct the overall activation distribution of a lymphatic chain, we employ the widely-used concept of “summation” in neurophysiology^64^. According to this concept, the resulting signal is formed by the simultaneous (spatial) and repeated (temporal) summation of multiple signals. In our model, this principle is applied to combine Gaussian functions generated at pacemakers, thus creating the overall spatiotemporal contraction distribution of a lymphatic network (Fig. 5c). Specifically, the activation parameter of each discretized node in the lymphatic chain is determined by the normalized algebraic summation of all signals at that node.

11 $$\overline{{\varvec{F}} }\left(z,t\right)=\sum_{i=1}^{K}{\overline{{\varvec{f}}} }_{i}(z,t)={\overline{{\varvec{f}}} }_{1}\left(z,t\right)+{\overline{{\varvec{f}}} }_{2}\left(z,t\right)+\dots +{\overline{{\varvec{f}}} }_{n}(z,t)$$

where $\overline{{\varvec{f}} }$ is a 2-D Gaussian function positioned at each pacemaker, while $K$ denotes the number of pacemakers, and $\overline{{\varvec{F}} }$ represents the overall activation function that encompasses the time–space domains. The value of ${t}_{act}$ for each node at each time step is calculated as,

12 $${{t}_{act}}_{j}^{i}=\frac{F\left({z}_{j},{t}_{i}\right)}{\Gamma }$$

where $\Gamma$ is the maximum activation parameter among all nodes during a contraction period.

#### Mathematical solver

An implicit finite difference scheme was employed to solve the governing equations, considering a given vascular geometry and material properties (see Supplementary). The collecting lymphangions are discretized with $dz=50 {\upmu m}$ and the time domain was divided into time steps of dt = 5 ms (Table 2). Using the discretized equations, a MATLAB routine was developed to calculate the general matrix of unknown pressures and flows at each time step. To assess the solver's robustness, conservation of mass was verified during each contraction cycle. This verification ensures that the cycle-averaged flow entering the first lymphangion remains constant along the chain. Furthermore, a sensitivity study was conducted to ensure the utilization of appropriate time and spatial steps.

**Table 2:** Simulation parameters.

Parameter	Value	Parameter	Value
${L}_{lymphangion} (\mathrm{mm})$	1^29, 67^	$dt (\mathrm{ms})$	5
$dz ({\upmu m})$	50	${P}_{inlet} ({\mathrm{cmH}}_{2}\mathrm{O})$	3.5
${N}_{lymphangion}$	3	${P}_{outlet} ({\mathrm{cmH}}_{2}\mathrm{O})$	4
${D}_{0} ({\upmu m})$	255	Lymph viscosity $({\mathrm{cmH}}_{2}\mathrm{O\,s})$	10^–5^^58^
Lymph density $(\frac{{\mathrm{cmH}}_{2}\mathrm{O }{\mathrm{s}}^{2}}{{\mathrm{cm}}^{2}})$	1070 × 10^–6^	Velocity profile constant (–)	2

### Supplementary metrics

#### Ejection fraction and fractional pump function

Ejection Fraction (EF) indicates the amount of lymph that exits the vessel during each contraction and is calculated using the average end-diastolic ($${A}_{EDA})$$ and end-systolic ($${A}_{ESA})$$ areas.

13 $$EF=\frac{{A}_{EDA}-{A}_{ESA}}{{A}_{EDA}}$$

And fractional pump function (FPF) is defined as follows:

14 $$FPF=Freq\times EF$$

#### Signal-to-noise ratio

Alongside magnitude, we propose that the smoothness of the WSS signal plays a crucial role in mechanotransduction events in lymphatic muscle cells. To explore this concept, we employed a metric known as the signal-to-noise ratio (SNR), derived from the mean-squared error (MSE) between a WSS signal and its filtered counterpart^65^.

15 $$SNR=\mathit{log}{\left(\frac{MS{E}_{0}}{MSE}\right)}^{2}$$

where $MSE$ and $MS{E}_{0}$ are calculated as follows

16 $$MSE=\sqrt{\frac{\sum_{n=1}^{N}{\left[wss(n)-\widehat{wss}(n)\right]}^{2}}{N}}$$

17 $$MS{E}_{0}=\sqrt{\frac{\sum_{n=1}^{N}{wss(n)}^{2}}{N}}$$

where $wss(n)$ represents the original signal containing noise, $N$ is the length of the signal, and $\widehat{wss}(n)$ corresponds to the denoised signal obtained by sliding a window of length *windowSize* along the data and calculating the average of the data within each window.

18 $$\widehat{wss}(n)=\frac{1}{windowSize}\left(x\left(n\right)+x\left(n-1\right)+\dots +x(n-(windowSize-1))\right)$$

#### Radial contraction velocity

Another metric is the radial contraction velocity, which is defined as the time derivative of the radius:

19 $$v=\frac{dr}{dt}$$

#### Valvular energy loss

In biomechanics, a crucial metric for studying valves is the energy loss caused by the inefficient performance of leaflets and the degree of turbulence in the flow field near the valves. Energy loss refers to the overall energy difference at the valve. The total mechanical energy per volume ($\Phi$) is defined as^66^.

20 $$\Phi =P+\frac{1}{2}\rho {u}^{2}$$

$P$ represents the luminal pressure, $\rho$ is the lymph density, and $u$ denotes the lymph axial velocity. The energy loss at a valve is determined by the difference in total mechanical energy before and after the valve:

21 $$\Delta \Phi ={\Phi }_{1}-{\Phi }_{2}$$

### Experimental data

To construct and validate the model using the experimental data, we utilized data from a study conducted by Gonzalez et al*.* that presents a comprehensive dataset on contractility in both wild-type and knock-out mouse strains^29^ (Table 3). The contraction propagation speed reported by Gonzalez et al*.* aligns with values previously documented by other researchers^9, 15^. The control group is based on data from the wild-type (Cx45^fx/fx^) (WT) mouse, while the abnormal group consists of the Smmhc-CreER^T2^; Cx45^fx/fx^ (TMX) (6–11 weeks) (KO) mouse line which is expected to exhibit notable differences in contraction patterns. As previously mentioned, the influence of contraction wave propagation direction poses an ongoing challenge^23, 24^. In order to assess the impact of propagation direction on lymphatic pumping in the KO mouse, three scenarios were considered. The first scenario assumes all contraction waves are antegrade, while the second scenario involves every other pacemaker generating a retrograde signal. In the third scenario, all pacemaking signals are retrograde. A diagram illustrating the directionality of pacemaking signals for each scenario is presented at the bottom of Fig. 1. The reason these scenarios were not explored for the WT model is that the high contraction propagation velocity renders the effect of wave propagation direction negligible.

**Table 3:** Contractility metrics used to prescribe the activation function.

Mouse line	Conduction speed (cm/s)	Number of initiation sites (1/cm)	Contraction frequency (1/min)	Contraction period (s)
WT	0.98	10	8.3	7.23
KO	0.06	31	15.8	3.80

The same parameters were used in three cases of the KO model.

### Supplementary Information

Supplementary Information.

## Data Availability

All data supporting the findings of this study are available within the article and the supplementary information files can be obtained from the corresponding author upon a reasonable request.
